# Bis[*N*-cyclohexyl-1-(2-{1-[(cyclohexyl­amino)carbonyl]cyclohexyl}-3,5-dioxo-1,2-oxazolidin-4-yl)cyclopentanecarbox­amide] monohydrate

**DOI:** 10.1107/S1600536811028868

**Published:** 2011-08-02

**Authors:** Enayatollah Sheikhhosseini, Fahime Vafadarnejad, Azizollah Habibi

**Affiliations:** a Faculty of Science, Department of Chemistry, University of Islamic Azad, Kerman Branch, Kerman, Iran; b Faculty of Chemistry, Tarbiat Moallem University, 49 Mofateh Avenue, Tehran, Iran

## Abstract

The reaction of cyclo­hexyl isocyanide and alkyl­idene Meldrum’s acid (systematic name 2,2-dimethyl-1,3-dioxane-4,6-dione) in the presence of cyclo­hexyl ketoxime and dichloro­methane as solvent resulted in the title compound, 2C_28_H_43_N_3_O_5_·H_2_O. One methyl­ene group of the cyclo­pentane ring was found to be disordered and was refined with occupancies 0.75:0.25. Intra­molecular N—H⋯O hydrogen bonds occur. The crystal structure is stabilized by inter­molecular N—H⋯O and O—H⋯O hydrogen bonds.

## Related literature

For the biological activity of isoxazoles, see: Conti *et al.* (1998 ▶); Kang *et al.* (2000 ▶); Ko *et al.* (1998 ▶); Mishra *et al.* (1998 ▶).
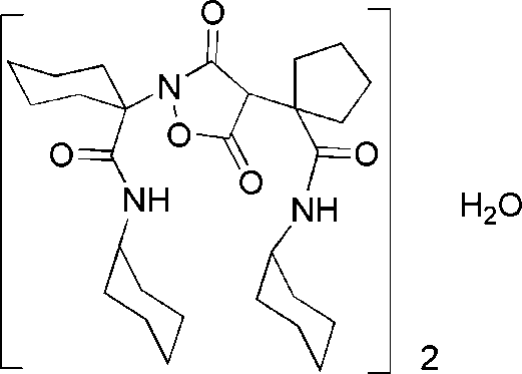


## Experimental

### 

#### Crystal data

2C_28_H_43_N_3_O_5_·H_2_O
*M* *_r_* = 1021.32
Triclinic, 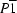

*a* = 11.3084 (6) Å
*b* = 12.3119 (7) Å
*c* = 20.7904 (11) Å
α = 102.686 (1)°
β = 103.104 (1)°
γ = 93.100 (1)°
*V* = 2733.9 (3) Å^3^
*Z* = 2
Mo *K*α radiation
μ = 0.09 mm^−1^
*T* = 100 K
0.60 × 0.50 × 0.10 mm

#### Data collection

Bruker APEXII CCD diffractometer
28848 measured reflections
11931 independent reflections
9099 reflections with *I* > 2σ(*I*)
*R*_int_ = 0.028

#### Refinement

*R*[*F* ^2^ > 2σ(*F* ^2^)] = 0.047
*wR*(*F* ^2^) = 0.120
*S* = 1.03
11931 reflections
675 parameters
H atoms treated by a mixture of independent and constrained refinement
Δρ_max_ = 0.38 e Å^−3^
Δρ_min_ = −0.25 e Å^−3^

### 

Data collection: *APEX2* (Bruker, 2005 ▶); cell refinement: *SAINT* (Bruker, 2005 ▶); data reduction: *SAINT*; program(s) used to solve structure: *SHELXS97* (Sheldrick, 2008 ▶); program(s) used to refine structure: *SHELXL97* (Sheldrick, 2008 ▶); molecular graphics: *SHELXTL* (Sheldrick, 2008 ▶); software used to prepare material for publication: *SHELXTL*.

## Supplementary Material

Crystal structure: contains datablock(s) I, global. DOI: 10.1107/S1600536811028868/vm2097sup1.cif

Structure factors: contains datablock(s) I. DOI: 10.1107/S1600536811028868/vm2097Isup2.hkl

Supplementary material file. DOI: 10.1107/S1600536811028868/vm2097Isup3.cml

Additional supplementary materials:  crystallographic information; 3D view; checkCIF report

## Figures and Tables

**Table 1:** Hydrogen-bond geometry (Å, °)

*D*—H⋯*A*	*D*—H	H⋯*A*	*D*⋯*A*	*D*—H⋯*A*
N2*A*—H2*AA*⋯O5*A*	0.88	2.09	2.9371 (18)	161
N3*A*—H3*AA*⋯O1*W*	0.88	2.00	2.8662 (19)	170
N2*B*—H2*BA*⋯O5*B*	0.88	2.10	2.9330 (18)	157
N3*B*—H3*BA*⋯O3*A*	0.88	2.07	2.9238 (17)	163
O1*W*—H1*WA*⋯O4*B*^i^	0.85 (3)	1.90 (3)	2.7433 (18)	169 (2)
O1*W*—H1*WB*⋯O4*A*^ii^	0.89 (2)	1.88 (2)	2.7569 (18)	170 (2)

Symmetry codes: (i) 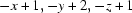; (ii) 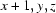.

**Fig. 1.**
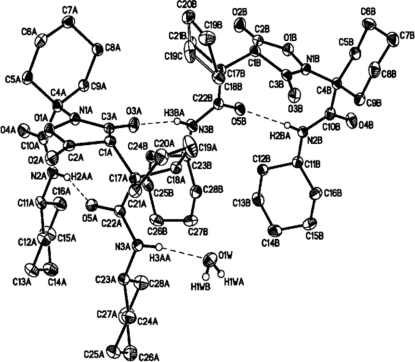
Molecular structure of the title compound with thermal elliposids drawn at 50% probability level. Hydrogen bonds are shown as dashed lines. Hydrogen atoms not involved inhydrogen bonds have been omitted for clarity.

**Fig. 2.**
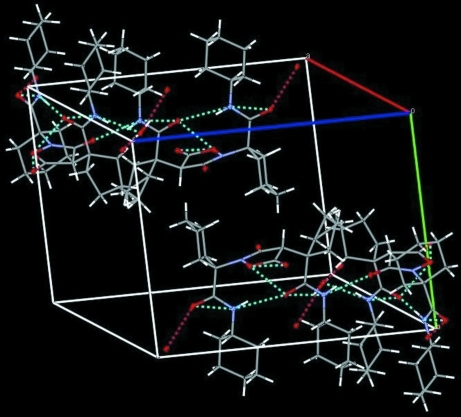
Unit cell packing diagram of the title compound. Hydrogen bonds are shown as dashed lines.

## supplementary crystallographic information

### Comment

Isoxazoles play an important role in heterocyclic chemistry and have served as versatile building blocks in organic synthesis. They have long been targeted in synthetic investigations for their known biological activities and pharmacological properties such as hypoglycemic, analgesic, anti-inflammatory and anti-bacterial activities (Conti *et al.*, 1998; Mishra *et al.*, 1998; Ko *et al.*, 1998; Kang *et al.*, 2000). The title compound was formed by reaction of cyclohexyl isocyanide and alkylidene Meldrum's acid in the presence of cyclohexyl ketoxime in dichloromethane as solvent. Fig. 1 shows the crystal structure of the title compound. The cyclohexyl and cyclopentyl rings have the normal shape (chair conformation for cyclohexyl and twisted envelope conformation for cyclopentyl ring), bond lengths and angles. Their planes align almost perpendicular in respect to the isoxazole core. The crystal structure is stabilized by N— H···O and O— H···O hydrogen bonds (Table 1, Fig. 2). Both molecules in the asymmetric unit interact by N3B—H3BA···O3A hydrogen bonding with D···A distance of 2.9238 (17) Å. There is also a N2B—H2BA···O5B intermolecular hydrogen bonding with D···A distance of 2.9330 (18) Å. The water molecule connects the molecules by N3A—H3AA···O1W, O1W—H1WA···O4B (-*x* + 1, -*y* + 2, -*z* + 1) and O1W—H1WB···O4A (*x* + 1, *y*, *z*) hydrogen bonds with D···A distances of 2.8662 (19), 2.7433 (18) and 2.7569 (18) Å respectively.

### Experimental

The title compound was prepared by reaction of cyclohexyl isocyanide (4 mmol, 0.439 g) and alkylidene Meldrum's acid (2 mmol, 0.421 g) in the presence of cyclohexyl ketoxime (2 mmol, 0.226 g) in dichloromethane at room temperature within four hours. After evaporating the solvent, a white powder was separated from the reaction mixture.Crystallization and single-crystal preparation was done from an ethanol solution. Colorless crystals were obtained after two weeks at room temperature.

### Refinement

The hydrogen atoms of the water molecule and NH groups were found in a difference Fourier synthesis. The hydrogen atoms of the water molecule were refined in an isotropic approximation. The other hydrogen atoms were refined using a riding model with the *U*_iso_(H) parameters equal to 1.2 *U*_eq_(Ci,Nj) and C—H = 0.99 and 1.00 Å, and N—H = 0.88 Å. In molecule B, atom C19 is disordered over two positions with occupancies 0.75/0.25.

### Crystal data

**Table:**

2C_28_H_43_N_3_O_5_·H_2_O	*Z* = 2
*M**_r_* = 1021.32	*F*(000) = 1108
Triclinic, *P*1	*D*_x_ = 1.241 Mg m^−^^3^
Hall symbol: -P 1	Mo *K*α radiation, λ = 0.71073 Å
*a* = 11.3084 (6) Å	Cell parameters from 7443 reflections
*b* = 12.3119 (7) Å	θ = 2.3–30.5°
*c* = 20.7904 (11) Å	µ = 0.09 mm^−^^1^
α = 102.686 (1)°	*T* = 100 K
β = 103.104 (1)°	Plate, colourless
γ = 93.100 (1)°	0.60 × 0.50 × 0.10 mm
*V* = 2733.9 (3) Å^3^	

### Data collection

**Table:**

Bruker APEXII CCD diffractometer	9099 reflections with *I* > 2σ(*I*)
Radiation source: fine-focus sealed tube	*R*_int_ = 0.028
graphite	θ_max_ = 27.0°, θ_min_ = 1.8°
φ and ω scans	*h* = −14→14
28848 measured reflections	*k* = −15→15
11931 independent reflections	*l* = −26→26

### Refinement

**Table:**

Refinement on *F*^2^	Primary atom site location: structure-invariant direct methods
Least-squares matrix: full	Secondary atom site location: difference Fourier map
*R*[*F*^2^ > 2σ(*F*^2^)] = 0.047	Hydrogen site location: mixed
*wR*(*F*^2^) = 0.120	H atoms treated by a mixture of independent and constrained refinement
*S* = 1.03	*w* = 1/[σ^2^(*F*_o_^2^) + (0.0534*P*)^2^ + 1.1858*P*] where *P* = (*F*_o_^2^ + 2*F*_c_^2^)/3
11931 reflections	(Δ/σ)_max_ = 0.001
675 parameters	Δρ_max_ = 0.38 e Å^−^^3^
0 restraints	Δρ_min_ = −0.25 e Å^−^^3^

### Special details

**Table:**

Geometry. All e.s.d.'s (except the e.s.d. in the dihedral angle between two l.s. planes) are estimated using the full covariance matrix. The cell e.s.d.'s are taken into account individually in the estimation of e.s.d.'s in distances, angles and torsion angles; correlations between e.s.d.'s in cell parameters are only used when they are defined by crystal symmetry. An approximate (isotropic) treatment of cell e.s.d.'s is used for estimating e.s.d.'s involving l.s. planes.
Refinement. Refinement of *F*^2^ against ALL reflections. The weighted *R*-factor *wR* and goodness of fit *S* are based on *F*^2^, conventional *R*-factors *R* are based on *F*, with *F* set to zero for negative *F*^2^. The threshold expression of *F*^2^ > σ(*F*^2^) is used only for calculating *R*-factors(gt) *etc*. and is not relevant to the choice of reflections for refinement. *R*-factors based on *F*^2^ are statistically about twice as large as those based on *F*, and *R*- factors based on ALL data will be even larger.

### Fractional atomic coordinates and isotropic or equivalent isotropic displacement parameters (Å^2^)

**Table:**

	*x*	*y*	*z*	*U*_iso_*/*U*_eq_	Occ. (<1)
O1A	−0.13320 (10)	0.65250 (9)	0.03789 (5)	0.0176 (2)	
O2A	0.01027 (10)	0.61354 (9)	−0.01804 (5)	0.0219 (2)	
O3A	−0.00419 (10)	0.72134 (10)	0.21203 (5)	0.0223 (3)	
O4A	−0.39218 (10)	0.84717 (10)	0.09932 (6)	0.0248 (3)	
O5A	0.05691 (10)	0.85482 (9)	0.09712 (6)	0.0195 (2)	
N1A	−0.13848 (12)	0.67986 (11)	0.10777 (6)	0.0161 (3)	
N2A	−0.19391 (12)	0.89376 (11)	0.10505 (7)	0.0193 (3)	
H2AA	−0.1201	0.8723	0.1095	0.023*	
N3A	0.25882 (12)	0.91251 (11)	0.13761 (7)	0.0190 (3)	
H3AA	0.3303	0.8948	0.1572	0.023*	
C1A	0.06124 (14)	0.64695 (12)	0.10753 (8)	0.0154 (3)	
H1AA	0.0758	0.5696	0.1127	0.018*	
C2A	−0.01422 (14)	0.63683 (12)	0.03600 (8)	0.0165 (3)	
C3A	−0.02645 (14)	0.68957 (12)	0.15035 (8)	0.0159 (3)	
C4A	−0.26030 (14)	0.70536 (13)	0.11588 (8)	0.0172 (3)	
C5A	−0.35683 (14)	0.61647 (14)	0.06433 (8)	0.0200 (3)	
H5AA	−0.4393	0.6368	0.0672	0.024*	
H5AB	−0.3486	0.6155	0.0178	0.024*	
C6A	−0.34373 (15)	0.49955 (14)	0.07719 (9)	0.0234 (4)	
H6AA	−0.4092	0.4455	0.0440	0.028*	
H6AB	−0.2643	0.4763	0.0702	0.028*	
C7A	−0.35121 (15)	0.49669 (14)	0.14915 (9)	0.0236 (4)	
H7AA	−0.4344	0.5102	0.1544	0.028*	
H7AB	−0.3357	0.4216	0.1569	0.028*	
C8A	−0.25824 (14)	0.58504 (14)	0.20171 (8)	0.0203 (3)	
H8AA	−0.1749	0.5650	0.2009	0.024*	
H8AB	−0.2701	0.5860	0.2476	0.024*	
C9A	−0.26971 (14)	0.70175 (13)	0.18846 (8)	0.0179 (3)	
H9AA	−0.2044	0.7554	0.2220	0.021*	
H9AB	−0.3493	0.7256	0.1950	0.021*	
C10A	−0.28653 (14)	0.82245 (13)	0.10540 (8)	0.0187 (3)	
C11A	−0.21330 (15)	1.00748 (13)	0.09738 (8)	0.0201 (3)	
H11A	−0.2916	1.0034	0.0625	0.024*	
C12A	−0.11029 (16)	1.05422 (14)	0.07207 (9)	0.0230 (3)	
H12A	−0.1073	1.0048	0.0281	0.028*	
H12B	−0.0315	1.0555	0.1050	0.028*	
C13A	−0.12769 (18)	1.17244 (14)	0.06302 (9)	0.0278 (4)	
H13A	−0.0572	1.2021	0.0488	0.033*	
H13B	−0.2022	1.1701	0.0267	0.033*	
C14A	−0.13910 (18)	1.24998 (15)	0.12894 (10)	0.0307 (4)	
H14A	−0.1558	1.3247	0.1210	0.037*	
H14B	−0.0611	1.2591	0.1638	0.037*	
C15A	−0.24185 (19)	1.20278 (15)	0.15483 (10)	0.0335 (4)	
H15A	−0.3210	1.2012	0.1221	0.040*	
H15B	−0.2445	1.2522	0.1989	0.040*	
C16A	−0.22361 (17)	1.08438 (15)	0.16395 (9)	0.0269 (4)	
H16A	−0.1484	1.0868	0.1998	0.032*	
H16B	−0.2935	1.0544	0.1786	0.032*	
C17A	0.18704 (13)	0.71710 (12)	0.12690 (8)	0.0156 (3)	
C18A	0.26142 (14)	0.70717 (13)	0.19718 (8)	0.0183 (3)	
H18A	0.3270	0.7702	0.2171	0.022*	
H18B	0.2079	0.7072	0.2288	0.022*	
C19A	0.3158 (2)	0.59538 (17)	0.18350 (10)	0.0363 (5)	
H19A	0.2692	0.5375	0.1970	0.044*	
H19B	0.4020	0.6038	0.2096	0.044*	
C20A	0.30729 (15)	0.56189 (14)	0.10647 (9)	0.0236 (4)	
H20A	0.3873	0.5435	0.0978	0.028*	
H20B	0.2460	0.4962	0.0839	0.028*	
C21A	0.26873 (14)	0.66455 (13)	0.08054 (8)	0.0187 (3)	
H21A	0.2224	0.6426	0.0323	0.022*	
H21B	0.3408	0.7175	0.0849	0.022*	
C22A	0.16267 (14)	0.83580 (13)	0.12053 (8)	0.0165 (3)	
C23A	0.25175 (15)	1.02484 (13)	0.12546 (8)	0.0189 (3)	
H23A	0.1742	1.0236	0.0906	0.023*	
C24A	0.35833 (16)	1.05665 (14)	0.09700 (9)	0.0242 (4)	
H24B	0.4363	1.0576	0.1305	0.029*	
H24C	0.3578	1.0000	0.0549	0.029*	
C25A	0.34905 (18)	1.17178 (15)	0.08134 (9)	0.0314 (4)	
H25A	0.4204	1.1923	0.0646	0.038*	
H25B	0.2744	1.1691	0.0451	0.038*	
C26A	0.34470 (18)	1.26019 (15)	0.14446 (10)	0.0338 (4)	
H26A	0.4235	1.2692	0.1787	0.041*	
H26B	0.3328	1.3329	0.1323	0.041*	
C27A	0.24173 (19)	1.22803 (15)	0.17496 (11)	0.0361 (5)	
H27A	0.1624	1.2289	0.1430	0.043*	
H27B	0.2455	1.2841	0.2178	0.043*	
C28A	0.24952 (17)	1.11198 (14)	0.18959 (9)	0.0269 (4)	
H28A	0.3245	1.1131	0.2254	0.032*	
H28B	0.1783	1.0918	0.2065	0.032*	
O1B	0.16762 (10)	0.67637 (9)	0.57514 (6)	0.0204 (2)	
O2B	−0.02284 (11)	0.63046 (11)	0.51393 (6)	0.0282 (3)	
O3B	0.36665 (10)	0.65175 (10)	0.46859 (6)	0.0238 (3)	
O4B	0.43163 (12)	0.91008 (10)	0.70259 (6)	0.0305 (3)	
O5B	0.14185 (11)	0.81491 (9)	0.47007 (5)	0.0231 (3)	
N1B	0.28750 (11)	0.67806 (11)	0.56210 (6)	0.0171 (3)	
N2B	0.32941 (12)	0.91170 (11)	0.59588 (7)	0.0189 (3)	
H2BA	0.2902	0.8715	0.5554	0.023*	
N3B	0.07309 (13)	0.81334 (11)	0.35903 (7)	0.0213 (3)	
H3BA	0.0514	0.7721	0.3171	0.026*	
C1B	0.14912 (15)	0.60000 (13)	0.45886 (8)	0.0187 (3)	
H1BA	0.1448	0.5164	0.4483	0.022*	
C2B	0.08379 (15)	0.63657 (13)	0.51466 (8)	0.0196 (3)	
C3B	0.28102 (14)	0.64675 (13)	0.49443 (8)	0.0177 (3)	
C4B	0.38224 (14)	0.73222 (13)	0.62359 (8)	0.0176 (3)	
C5B	0.36582 (16)	0.68078 (14)	0.68243 (8)	0.0236 (4)	
H5BA	0.4218	0.7245	0.7253	0.028*	
H5BB	0.2811	0.6859	0.6874	0.028*	
C6B	0.39141 (18)	0.55844 (15)	0.67121 (9)	0.0305 (4)	
H6BA	0.3836	0.5303	0.7113	0.037*	
H6BB	0.3300	0.5131	0.6311	0.037*	
C7B	0.51869 (17)	0.54440 (15)	0.66002 (9)	0.0309 (4)	
H7BA	0.5806	0.5837	0.7017	0.037*	
H7BB	0.5307	0.4639	0.6508	0.037*	
C8B	0.53603 (16)	0.59126 (15)	0.60090 (9)	0.0268 (4)	
H8BA	0.4804	0.5463	0.5583	0.032*	
H8BB	0.6210	0.5857	0.5965	0.032*	
C9B	0.51007 (15)	0.71315 (14)	0.61091 (8)	0.0215 (3)	
H9BA	0.5170	0.7393	0.5700	0.026*	
H9BB	0.5729	0.7591	0.6501	0.026*	
C10B	0.37977 (15)	0.86003 (14)	0.64358 (8)	0.0204 (3)	
C11B	0.33796 (15)	1.03416 (13)	0.60937 (8)	0.0196 (3)	
H11B	0.3238	1.0636	0.6555	0.024*	
C12B	0.23958 (15)	1.06971 (13)	0.55735 (8)	0.0209 (3)	
H12C	0.1581	1.0390	0.5594	0.025*	
H12D	0.2503	1.0391	0.5111	0.025*	
C13B	0.24687 (17)	1.19743 (14)	0.57150 (9)	0.0284 (4)	
H13C	0.1835	1.2195	0.5368	0.034*	
H13D	0.2312	1.2278	0.6166	0.034*	
C14B	0.37301 (19)	1.24610 (15)	0.56991 (10)	0.0351 (4)	
H14C	0.3860	1.2198	0.5238	0.042*	
H14D	0.3774	1.3288	0.5803	0.042*	
C15B	0.47295 (18)	1.21077 (15)	0.62142 (11)	0.0349 (4)	
H15C	0.4647	1.2432	0.6680	0.042*	
H15D	0.5539	1.2400	0.6179	0.042*	
C16B	0.46468 (16)	1.08332 (14)	0.60890 (10)	0.0271 (4)	
H16C	0.5269	1.0624	0.6446	0.032*	
H16D	0.4822	1.0515	0.5644	0.032*	
C17B	0.09761 (14)	0.63456 (13)	0.39177 (8)	0.0178 (3)	
C18B	0.16391 (16)	0.58323 (13)	0.33668 (8)	0.0222 (3)	
H18C	0.2496	0.5840	0.3561	0.027*	
H18D	0.1538	0.6238	0.3016	0.027*	
C19B	0.1021 (3)	0.4634 (2)	0.30824 (15)	0.0298 (6)	0.75
H19C	0.0970	0.4395	0.2589	0.036*	0.75
H19D	0.1493	0.4115	0.3314	0.036*	0.75
C19C	0.0770 (8)	0.4970 (7)	0.2808 (4)	0.034 (2)	0.25
H19E	0.1184	0.4318	0.2637	0.040*	0.25
H19F	0.0392	0.5293	0.2425	0.040*	0.25
C20B	−0.02488 (16)	0.46242 (14)	0.32074 (10)	0.0288 (4)	
H20C	−0.0362	0.4100	0.3474	0.035*	
H20D	−0.0856	0.4416	0.2783	0.035*	
C21B	−0.03579 (15)	0.58055 (14)	0.35886 (8)	0.0223 (3)	
H21C	−0.0812	0.5786	0.3942	0.027*	
H21D	−0.0788	0.6232	0.3272	0.027*	
C22B	0.10709 (14)	0.76261 (13)	0.40977 (8)	0.0182 (3)	
C23B	0.07040 (14)	0.93452 (13)	0.37026 (8)	0.0182 (3)	
H23B	0.0716	0.9650	0.4192	0.022*	
C24B	−0.04667 (15)	0.96237 (13)	0.32703 (8)	0.0197 (3)	
H24A	−0.1184	0.9236	0.3357	0.024*	
H24D	−0.0485	0.9362	0.2782	0.024*	
C25B	−0.05234 (16)	1.08885 (14)	0.34468 (9)	0.0233 (4)	
H25C	−0.1285	1.1070	0.3167	0.028*	
H25D	−0.0534	1.1143	0.3931	0.028*	
C26B	0.05742 (17)	1.14980 (14)	0.33163 (9)	0.0282 (4)	
H26C	0.0551	1.2316	0.3462	0.034*	
H26D	0.0530	1.1308	0.2823	0.034*	
C27B	0.17706 (17)	1.11818 (15)	0.36985 (10)	0.0300 (4)	
H27C	0.1877	1.1485	0.4193	0.036*	
H27D	0.2457	1.1529	0.3564	0.036*	
C28B	0.18147 (15)	0.99154 (14)	0.35580 (9)	0.0251 (4)	
H28C	0.1829	0.9621	0.3077	0.030*	
H28D	0.2571	0.9746	0.3849	0.030*	
O1W	0.50041 (11)	0.88475 (10)	0.20760 (7)	0.0232 (3)	
H1WA	0.531 (2)	0.946 (2)	0.2367 (13)	0.053 (7)*	
H1WB	0.540 (2)	0.881 (2)	0.1749 (13)	0.053 (7)*	

### Atomic displacement parameters (Å^2^)

**Table:**

	*U*^11^	*U*^22^	*U*^33^	*U*^12^	*U*^13^	*U*^23^
O1A	0.0175 (5)	0.0229 (6)	0.0116 (5)	0.0020 (4)	0.0033 (4)	0.0031 (4)
O2A	0.0243 (6)	0.0244 (6)	0.0163 (6)	0.0006 (5)	0.0067 (5)	0.0020 (5)
O3A	0.0181 (6)	0.0337 (7)	0.0131 (5)	0.0012 (5)	0.0030 (4)	0.0029 (5)
O4A	0.0194 (6)	0.0300 (7)	0.0287 (6)	0.0085 (5)	0.0076 (5)	0.0114 (5)
O5A	0.0164 (5)	0.0186 (6)	0.0246 (6)	0.0033 (4)	0.0043 (5)	0.0075 (5)
N1A	0.0168 (6)	0.0200 (7)	0.0119 (6)	0.0024 (5)	0.0047 (5)	0.0032 (5)
N2A	0.0185 (7)	0.0204 (7)	0.0212 (7)	0.0060 (5)	0.0052 (5)	0.0081 (6)
N3A	0.0164 (6)	0.0171 (7)	0.0233 (7)	0.0016 (5)	0.0020 (5)	0.0075 (5)
C1A	0.0172 (7)	0.0126 (7)	0.0169 (7)	0.0022 (6)	0.0048 (6)	0.0039 (6)
C2A	0.0175 (7)	0.0124 (7)	0.0191 (8)	0.0000 (6)	0.0036 (6)	0.0039 (6)
C3A	0.0156 (7)	0.0150 (7)	0.0176 (8)	0.0008 (6)	0.0036 (6)	0.0058 (6)
C4A	0.0132 (7)	0.0219 (8)	0.0169 (8)	0.0036 (6)	0.0036 (6)	0.0054 (6)
C5A	0.0149 (7)	0.0258 (8)	0.0178 (8)	0.0006 (6)	0.0023 (6)	0.0042 (6)
C6A	0.0175 (8)	0.0231 (8)	0.0257 (9)	−0.0007 (6)	0.0020 (7)	0.0017 (7)
C7A	0.0172 (8)	0.0223 (8)	0.0316 (9)	0.0007 (6)	0.0041 (7)	0.0093 (7)
C8A	0.0170 (8)	0.0256 (8)	0.0204 (8)	0.0021 (6)	0.0046 (6)	0.0095 (7)
C9A	0.0157 (7)	0.0222 (8)	0.0169 (8)	0.0032 (6)	0.0052 (6)	0.0058 (6)
C10A	0.0193 (8)	0.0238 (8)	0.0142 (7)	0.0053 (6)	0.0047 (6)	0.0055 (6)
C11A	0.0234 (8)	0.0202 (8)	0.0185 (8)	0.0066 (6)	0.0051 (7)	0.0074 (6)
C12A	0.0292 (9)	0.0212 (8)	0.0223 (8)	0.0060 (7)	0.0108 (7)	0.0075 (7)
C13A	0.0364 (10)	0.0241 (9)	0.0283 (9)	0.0079 (8)	0.0118 (8)	0.0125 (7)
C14A	0.0404 (11)	0.0197 (9)	0.0355 (10)	0.0061 (8)	0.0138 (9)	0.0084 (8)
C15A	0.0458 (12)	0.0236 (9)	0.0357 (10)	0.0109 (8)	0.0204 (9)	0.0043 (8)
C16A	0.0354 (10)	0.0263 (9)	0.0220 (9)	0.0055 (8)	0.0122 (8)	0.0059 (7)
C17A	0.0145 (7)	0.0161 (7)	0.0167 (7)	0.0013 (6)	0.0049 (6)	0.0039 (6)
C18A	0.0169 (7)	0.0200 (8)	0.0179 (8)	0.0005 (6)	0.0031 (6)	0.0059 (6)
C19A	0.0468 (12)	0.0377 (11)	0.0269 (10)	0.0226 (9)	0.0064 (9)	0.0111 (8)
C20A	0.0202 (8)	0.0234 (9)	0.0274 (9)	0.0077 (7)	0.0058 (7)	0.0055 (7)
C21A	0.0161 (7)	0.0206 (8)	0.0205 (8)	0.0037 (6)	0.0069 (6)	0.0042 (6)
C22A	0.0190 (8)	0.0178 (8)	0.0141 (7)	0.0031 (6)	0.0066 (6)	0.0040 (6)
C23A	0.0203 (8)	0.0158 (7)	0.0201 (8)	0.0012 (6)	0.0026 (6)	0.0056 (6)
C24A	0.0281 (9)	0.0227 (8)	0.0236 (9)	−0.0007 (7)	0.0091 (7)	0.0071 (7)
C25A	0.0360 (10)	0.0290 (10)	0.0297 (10)	−0.0048 (8)	0.0033 (8)	0.0151 (8)
C26A	0.0391 (11)	0.0187 (9)	0.0373 (11)	−0.0034 (8)	−0.0037 (9)	0.0084 (8)
C27A	0.0428 (11)	0.0195 (9)	0.0413 (11)	0.0061 (8)	0.0080 (9)	−0.0007 (8)
C28A	0.0328 (10)	0.0222 (9)	0.0267 (9)	0.0033 (7)	0.0118 (8)	0.0031 (7)
O1B	0.0177 (6)	0.0252 (6)	0.0191 (6)	0.0025 (5)	0.0061 (5)	0.0050 (5)
O2B	0.0211 (6)	0.0377 (7)	0.0260 (6)	0.0010 (5)	0.0055 (5)	0.0085 (5)
O3B	0.0207 (6)	0.0306 (7)	0.0213 (6)	0.0038 (5)	0.0076 (5)	0.0055 (5)
O4B	0.0400 (7)	0.0223 (6)	0.0211 (6)	0.0064 (5)	−0.0040 (5)	−0.0007 (5)
O5B	0.0327 (7)	0.0167 (6)	0.0159 (6)	0.0025 (5)	−0.0006 (5)	0.0021 (4)
N1B	0.0146 (6)	0.0194 (7)	0.0170 (6)	0.0017 (5)	0.0037 (5)	0.0037 (5)
N2B	0.0214 (7)	0.0149 (6)	0.0178 (7)	0.0008 (5)	0.0023 (5)	0.0015 (5)
N3B	0.0317 (8)	0.0158 (7)	0.0142 (6)	0.0036 (6)	0.0020 (6)	0.0027 (5)
C1B	0.0231 (8)	0.0144 (7)	0.0174 (8)	0.0009 (6)	0.0025 (6)	0.0039 (6)
C2B	0.0203 (8)	0.0181 (8)	0.0209 (8)	0.0012 (6)	0.0043 (6)	0.0066 (6)
C3B	0.0209 (8)	0.0146 (7)	0.0183 (8)	0.0039 (6)	0.0042 (6)	0.0053 (6)
C4B	0.0198 (8)	0.0175 (8)	0.0134 (7)	0.0016 (6)	0.0013 (6)	0.0020 (6)
C5B	0.0271 (9)	0.0280 (9)	0.0153 (8)	−0.0009 (7)	0.0035 (7)	0.0067 (7)
C6B	0.0389 (10)	0.0266 (9)	0.0248 (9)	−0.0067 (8)	0.0020 (8)	0.0122 (7)
C7B	0.0354 (10)	0.0197 (9)	0.0307 (10)	0.0037 (7)	−0.0047 (8)	0.0046 (7)
C8B	0.0233 (9)	0.0256 (9)	0.0272 (9)	0.0071 (7)	0.0012 (7)	0.0017 (7)
C9B	0.0188 (8)	0.0233 (8)	0.0211 (8)	0.0011 (6)	0.0025 (6)	0.0048 (7)
C10B	0.0197 (8)	0.0204 (8)	0.0188 (8)	0.0021 (6)	0.0033 (6)	0.0013 (6)
C11B	0.0217 (8)	0.0155 (8)	0.0199 (8)	0.0021 (6)	0.0048 (6)	0.0008 (6)
C12B	0.0227 (8)	0.0175 (8)	0.0210 (8)	0.0007 (6)	0.0038 (7)	0.0034 (6)
C13B	0.0373 (10)	0.0191 (9)	0.0265 (9)	0.0070 (7)	0.0037 (8)	0.0036 (7)
C14B	0.0452 (12)	0.0177 (9)	0.0381 (11)	−0.0055 (8)	0.0026 (9)	0.0073 (8)
C15B	0.0324 (10)	0.0252 (10)	0.0423 (11)	−0.0084 (8)	0.0029 (9)	0.0073 (8)
C16B	0.0227 (9)	0.0242 (9)	0.0327 (10)	−0.0005 (7)	0.0058 (7)	0.0052 (7)
C17B	0.0210 (8)	0.0157 (7)	0.0148 (7)	0.0011 (6)	0.0002 (6)	0.0038 (6)
C18B	0.0287 (9)	0.0204 (8)	0.0179 (8)	0.0038 (7)	0.0068 (7)	0.0042 (6)
C19B	0.0379 (16)	0.0201 (13)	0.0310 (15)	0.0027 (11)	0.0130 (13)	0.0006 (11)
C19C	0.046 (5)	0.026 (4)	0.018 (4)	0.023 (4)	−0.006 (4)	−0.009 (3)
C20B	0.0292 (9)	0.0179 (8)	0.0325 (10)	−0.0006 (7)	−0.0019 (8)	0.0025 (7)
C21B	0.0210 (8)	0.0225 (8)	0.0210 (8)	0.0000 (7)	0.0010 (7)	0.0050 (7)
C22B	0.0178 (7)	0.0185 (8)	0.0171 (8)	0.0013 (6)	0.0027 (6)	0.0039 (6)
C23B	0.0223 (8)	0.0156 (7)	0.0159 (7)	0.0023 (6)	0.0027 (6)	0.0040 (6)
C24B	0.0210 (8)	0.0206 (8)	0.0174 (8)	0.0023 (6)	0.0041 (6)	0.0052 (6)
C25B	0.0288 (9)	0.0235 (9)	0.0208 (8)	0.0105 (7)	0.0083 (7)	0.0079 (7)
C26B	0.0400 (10)	0.0190 (8)	0.0296 (9)	0.0040 (7)	0.0124 (8)	0.0101 (7)
C27B	0.0298 (9)	0.0250 (9)	0.0356 (10)	−0.0056 (7)	0.0082 (8)	0.0096 (8)
C28B	0.0198 (8)	0.0276 (9)	0.0288 (9)	0.0034 (7)	0.0053 (7)	0.0093 (7)
O1W	0.0209 (6)	0.0200 (6)	0.0262 (6)	0.0006 (5)	0.0060 (5)	0.0003 (5)

### Geometric parameters (Å, °)

**Table:**

O1A—C2A	1.3777 (18)	O3B—C3B	1.2141 (19)
O1A—N1A	1.4332 (16)	O4B—C10B	1.237 (2)
O2A—C2A	1.1955 (19)	O5B—C22B	1.2391 (19)
O3A—C3A	1.2179 (18)	N1B—C3B	1.358 (2)
O4A—C10A	1.2344 (19)	N1B—C4B	1.4677 (19)
O5A—C22A	1.2403 (18)	N2B—C10B	1.331 (2)
N1A—C3A	1.3534 (19)	N2B—C11B	1.465 (2)
N1A—C4A	1.4675 (19)	N2B—H2BA	0.8800
N2A—C10A	1.332 (2)	N3B—C22B	1.335 (2)
N2A—C11A	1.466 (2)	N3B—C23B	1.462 (2)
N2A—H2AA	0.8800	N3B—H3BA	0.8800
N3A—C22A	1.329 (2)	C1B—C2B	1.513 (2)
N3A—C23A	1.4625 (19)	C1B—C3B	1.524 (2)
N3A—H3AA	0.8800	C1B—C17B	1.544 (2)
C1A—C2A	1.512 (2)	C1B—H1BA	1.0000
C1A—C3A	1.518 (2)	C4B—C5B	1.537 (2)
C1A—C17A	1.545 (2)	C4B—C10B	1.540 (2)
C1A—H1AA	1.0000	C4B—C9B	1.548 (2)
C4A—C5A	1.537 (2)	C5B—C6B	1.526 (2)
C4A—C10A	1.539 (2)	C5B—H5BA	0.9900
C4A—C9A	1.547 (2)	C5B—H5BB	0.9900
C5A—C6A	1.530 (2)	C6B—C7B	1.522 (3)
C5A—H5AA	0.9900	C6B—H6BA	0.9900
C5A—H5AB	0.9900	C6B—H6BB	0.9900
C6A—C7A	1.525 (2)	C7B—C8B	1.513 (3)
C6A—H6AA	0.9900	C7B—H7BA	0.9900
C6A—H6AB	0.9900	C7B—H7BB	0.9900
C7A—C8A	1.522 (2)	C8B—C9B	1.523 (2)
C7A—H7AA	0.9900	C8B—H8BA	0.9900
C7A—H7AB	0.9900	C8B—H8BB	0.9900
C8A—C9A	1.528 (2)	C9B—H9BA	0.9900
C8A—H8AA	0.9900	C9B—H9BB	0.9900
C8A—H8AB	0.9900	C11B—C12B	1.521 (2)
C9A—H9AA	0.9900	C11B—C16B	1.528 (2)
C9A—H9AB	0.9900	C11B—H11B	1.0000
C11A—C12A	1.518 (2)	C12B—C13B	1.529 (2)
C11A—C16A	1.528 (2)	C12B—H12C	0.9900
C11A—H11A	1.0000	C12B—H12D	0.9900
C12A—C13A	1.525 (2)	C13B—C14B	1.527 (3)
C12A—H12A	0.9900	C13B—H13C	0.9900
C12A—H12B	0.9900	C13B—H13D	0.9900
C13A—C14A	1.525 (2)	C14B—C15B	1.524 (3)
C13A—H13A	0.9900	C14B—H14C	0.9900
C13A—H13B	0.9900	C14B—H14D	0.9900
C14A—C15A	1.525 (3)	C15B—C16B	1.527 (2)
C14A—H14A	0.9900	C15B—H15C	0.9900
C14A—H14B	0.9900	C15B—H15D	0.9900
C15A—C16A	1.530 (2)	C16B—H16C	0.9900
C15A—H15A	0.9900	C16B—H16D	0.9900
C15A—H15B	0.9900	C17B—C22B	1.530 (2)
C16A—H16A	0.9900	C17B—C18B	1.548 (2)
C16A—H16B	0.9900	C17B—C21B	1.552 (2)
C17A—C22A	1.530 (2)	C18B—C19C	1.506 (8)
C17A—C18A	1.546 (2)	C18B—C19B	1.528 (3)
C17A—C21A	1.548 (2)	C18B—H18C	0.9600
C18A—C19A	1.534 (2)	C18B—H18D	0.9602
C18A—H18A	0.9900	C19B—C20B	1.516 (3)
C18A—H18B	0.9900	C19B—H19C	0.9900
C19A—C20A	1.543 (2)	C19B—H19D	0.9900
C19A—H19A	0.9900	C19C—C20B	1.653 (10)
C19A—H19B	0.9900	C19C—H19E	0.9900
C20A—C21A	1.527 (2)	C19C—H19F	0.9900
C20A—H20A	0.9900	C20B—C21B	1.522 (2)
C20A—H20B	0.9900	C20B—H20C	0.9600
C21A—H21A	0.9900	C20B—H20D	0.9598
C21A—H21B	0.9900	C21B—H21C	0.9900
C23A—C28A	1.523 (2)	C21B—H21D	0.9900
C23A—C24A	1.527 (2)	C23B—C24B	1.524 (2)
C23A—H23A	1.0000	C23B—C28B	1.526 (2)
C24A—C25A	1.526 (2)	C23B—H23B	1.0000
C24A—H24B	0.9900	C24B—C25B	1.528 (2)
C24A—H24C	0.9900	C24B—H24A	0.9900
C25A—C26A	1.522 (3)	C24B—H24D	0.9900
C25A—H25A	0.9900	C25B—C26B	1.523 (2)
C25A—H25B	0.9900	C25B—H25C	0.9900
C26A—C27A	1.520 (3)	C25B—H25D	0.9900
C26A—H26A	0.9900	C26B—C27B	1.523 (3)
C26A—H26B	0.9900	C26B—H26C	0.9900
C27A—C28A	1.528 (2)	C26B—H26D	0.9900
C27A—H27A	0.9900	C27B—C28B	1.528 (2)
C27A—H27B	0.9900	C27B—H27C	0.9900
C28A—H28A	0.9900	C27B—H27D	0.9900
C28A—H28B	0.9900	C28B—H28C	0.9900
O1B—C2B	1.3633 (19)	C28B—H28D	0.9900
O1B—N1B	1.4415 (16)	O1W—H1WA	0.86 (3)
O2B—C2B	1.200 (2)	O1W—H1WB	0.89 (3)
			
C2A—O1A—N1A	107.90 (11)	C22B—N3B—H3BA	118.6
C3A—N1A—O1A	111.76 (12)	C23B—N3B—H3BA	118.6
C3A—N1A—C4A	135.16 (13)	C2B—C1B—C3B	102.17 (12)
O1A—N1A—C4A	112.43 (11)	C2B—C1B—C17B	115.77 (13)
C10A—N2A—C11A	120.88 (13)	C3B—C1B—C17B	115.01 (13)
C10A—N2A—H2AA	119.6	C2B—C1B—H1BA	107.8
C11A—N2A—H2AA	119.6	C3B—C1B—H1BA	107.8
C22A—N3A—C23A	123.23 (13)	C17B—C1B—H1BA	107.8
C22A—N3A—H3AA	118.4	O2B—C2B—O1B	119.02 (15)
C23A—N3A—H3AA	118.4	O2B—C2B—C1B	131.38 (15)
C2A—C1A—C3A	102.38 (12)	O1B—C2B—C1B	109.47 (13)
C2A—C1A—C17A	115.89 (12)	O3B—C3B—N1B	125.39 (15)
C3A—C1A—C17A	115.38 (12)	O3B—C3B—C1B	127.42 (14)
C2A—C1A—H1AA	107.6	N1B—C3B—C1B	107.13 (13)
C3A—C1A—H1AA	107.6	N1B—C4B—C5B	109.19 (13)
C17A—C1A—H1AA	107.6	N1B—C4B—C10B	112.50 (12)
O2A—C2A—O1A	118.70 (14)	C5B—C4B—C10B	109.95 (13)
O2A—C2A—C1A	132.03 (14)	N1B—C4B—C9B	109.71 (12)
O1A—C2A—C1A	109.15 (12)	C5B—C4B—C9B	109.15 (13)
O3A—C3A—N1A	124.94 (14)	C10B—C4B—C9B	106.27 (13)
O3A—C3A—C1A	127.48 (14)	C6B—C5B—C4B	112.34 (14)
N1A—C3A—C1A	107.48 (13)	C6B—C5B—H5BA	109.1
N1A—C4A—C5A	109.09 (12)	C4B—C5B—H5BA	109.1
N1A—C4A—C10A	111.89 (12)	C6B—C5B—H5BB	109.1
C5A—C4A—C10A	109.56 (12)	C4B—C5B—H5BB	109.1
N1A—C4A—C9A	109.53 (12)	H5BA—C5B—H5BB	107.9
C5A—C4A—C9A	108.68 (13)	C7B—C6B—C5B	111.64 (14)
C10A—C4A—C9A	108.04 (12)	C7B—C6B—H6BA	109.3
C6A—C5A—C4A	111.94 (13)	C5B—C6B—H6BA	109.3
C6A—C5A—H5AA	109.2	C7B—C6B—H6BB	109.3
C4A—C5A—H5AA	109.2	C5B—C6B—H6BB	109.3
C6A—C5A—H5AB	109.2	H6BA—C6B—H6BB	108.0
C4A—C5A—H5AB	109.2	C8B—C7B—C6B	110.84 (14)
H5AA—C5A—H5AB	107.9	C8B—C7B—H7BA	109.5
C7A—C6A—C5A	111.67 (14)	C6B—C7B—H7BA	109.5
C7A—C6A—H6AA	109.3	C8B—C7B—H7BB	109.5
C5A—C6A—H6AA	109.3	C6B—C7B—H7BB	109.5
C7A—C6A—H6AB	109.3	H7BA—C7B—H7BB	108.1
C5A—C6A—H6AB	109.3	C7B—C8B—C9B	111.20 (14)
H6AA—C6A—H6AB	107.9	C7B—C8B—H8BA	109.4
C8A—C7A—C6A	110.95 (13)	C9B—C8B—H8BA	109.4
C8A—C7A—H7AA	109.4	C7B—C8B—H8BB	109.4
C6A—C7A—H7AA	109.4	C9B—C8B—H8BB	109.4
C8A—C7A—H7AB	109.4	H8BA—C8B—H8BB	108.0
C6A—C7A—H7AB	109.4	C8B—C9B—C4B	113.51 (14)
H7AA—C7A—H7AB	108.0	C8B—C9B—H9BA	108.9
C7A—C8A—C9A	111.98 (13)	C4B—C9B—H9BA	108.9
C7A—C8A—H8AA	109.2	C8B—C9B—H9BB	108.9
C9A—C8A—H8AA	109.2	C4B—C9B—H9BB	108.9
C7A—C8A—H8AB	109.2	H9BA—C9B—H9BB	107.7
C9A—C8A—H8AB	109.2	O4B—C10B—N2B	123.39 (15)
H8AA—C8A—H8AB	107.9	O4B—C10B—C4B	117.72 (14)
C8A—C9A—C4A	112.35 (13)	N2B—C10B—C4B	118.74 (14)
C8A—C9A—H9AA	109.1	N2B—C11B—C12B	110.02 (13)
C4A—C9A—H9AA	109.1	N2B—C11B—C16B	111.03 (13)
C8A—C9A—H9AB	109.1	C12B—C11B—C16B	110.74 (14)
C4A—C9A—H9AB	109.1	N2B—C11B—H11B	108.3
H9AA—C9A—H9AB	107.9	C12B—C11B—H11B	108.3
O4A—C10A—N2A	122.80 (15)	C16B—C11B—H11B	108.3
O4A—C10A—C4A	118.48 (14)	C11B—C12B—C13B	110.40 (13)
N2A—C10A—C4A	118.70 (13)	C11B—C12B—H12C	109.6
N2A—C11A—C12A	109.72 (13)	C13B—C12B—H12C	109.6
N2A—C11A—C16A	111.55 (13)	C11B—C12B—H12D	109.6
C12A—C11A—C16A	110.71 (14)	C13B—C12B—H12D	109.6
N2A—C11A—H11A	108.3	H12C—C12B—H12D	108.1
C12A—C11A—H11A	108.3	C14B—C13B—C12B	110.15 (15)
C16A—C11A—H11A	108.3	C14B—C13B—H13C	109.6
C11A—C12A—C13A	111.27 (14)	C12B—C13B—H13C	109.6
C11A—C12A—H12A	109.4	C14B—C13B—H13D	109.6
C13A—C12A—H12A	109.4	C12B—C13B—H13D	109.6
C11A—C12A—H12B	109.4	H13C—C13B—H13D	108.1
C13A—C12A—H12B	109.4	C15B—C14B—C13B	110.93 (15)
H12A—C12A—H12B	108.0	C15B—C14B—H14C	109.5
C14A—C13A—C12A	111.03 (14)	C13B—C14B—H14C	109.5
C14A—C13A—H13A	109.4	C15B—C14B—H14D	109.5
C12A—C13A—H13A	109.4	C13B—C14B—H14D	109.5
C14A—C13A—H13B	109.4	H14C—C14B—H14D	108.0
C12A—C13A—H13B	109.4	C14B—C15B—C16B	110.77 (15)
H13A—C13A—H13B	108.0	C14B—C15B—H15C	109.5
C13A—C14A—C15A	110.85 (15)	C16B—C15B—H15C	109.5
C13A—C14A—H14A	109.5	C14B—C15B—H15D	109.5
C15A—C14A—H14A	109.5	C16B—C15B—H15D	109.5
C13A—C14A—H14B	109.5	H15C—C15B—H15D	108.1
C15A—C14A—H14B	109.5	C15B—C16B—C11B	111.12 (14)
H14A—C14A—H14B	108.1	C15B—C16B—H16C	109.4
C14A—C15A—C16A	111.26 (15)	C11B—C16B—H16C	109.4
C14A—C15A—H15A	109.4	C15B—C16B—H16D	109.4
C16A—C15A—H15A	109.4	C11B—C16B—H16D	109.4
C14A—C15A—H15B	109.4	H16C—C16B—H16D	108.0
C16A—C15A—H15B	109.4	C22B—C17B—C1B	106.34 (12)
H15A—C15A—H15B	108.0	C22B—C17B—C18B	114.76 (13)
C11A—C16A—C15A	110.48 (14)	C1B—C17B—C18B	110.93 (13)
C11A—C16A—H16A	109.6	C22B—C17B—C21B	112.20 (13)
C15A—C16A—H16A	109.6	C1B—C17B—C21B	110.94 (13)
C11A—C16A—H16B	109.6	C18B—C17B—C21B	101.75 (13)
C15A—C16A—H16B	109.6	C19C—C18B—C19B	30.1 (3)
H16A—C16A—H16B	108.1	C19C—C18B—C17B	109.7 (4)
C22A—C17A—C1A	106.74 (12)	C19B—C18B—C17B	104.39 (15)
C22A—C17A—C18A	115.76 (13)	C19C—C18B—H18C	130.2
C1A—C17A—C18A	110.46 (12)	C19B—C18B—H18C	111.0
C22A—C17A—C21A	112.27 (12)	C17B—C18B—H18C	111.0
C1A—C17A—C21A	110.58 (12)	C19C—C18B—H18D	81.5
C18A—C17A—C21A	101.02 (12)	C19B—C18B—H18D	110.7
C19A—C18A—C17A	105.19 (13)	C17B—C18B—H18D	110.8
C19A—C18A—H18A	110.7	H18C—C18B—H18D	108.9
C17A—C18A—H18A	110.7	C20B—C19B—C18B	107.17 (18)
C19A—C18A—H18B	110.7	C20B—C19B—H19C	110.3
C17A—C18A—H18B	110.7	C18B—C19B—H19C	110.3
H18A—C18A—H18B	108.8	C20B—C19B—H19D	110.3
C18A—C19A—C20A	106.64 (14)	C18B—C19B—H19D	110.3
C18A—C19A—H19A	110.4	H19C—C19B—H19D	108.5
C20A—C19A—H19A	110.4	C18B—C19C—C20B	101.5 (5)
C18A—C19A—H19B	110.4	C18B—C19C—H19E	111.5
C20A—C19A—H19B	110.4	C20B—C19C—H19E	111.5
H19A—C19A—H19B	108.6	C18B—C19C—H19F	111.5
C21A—C20A—C19A	104.93 (13)	C20B—C19C—H19F	111.5
C21A—C20A—H20A	110.8	H19E—C19C—H19F	109.3
C19A—C20A—H20A	110.8	C19B—C20B—C21B	106.71 (15)
C21A—C20A—H20B	110.8	C19B—C20B—C19C	28.3 (3)
C19A—C20A—H20B	110.8	C21B—C20B—C19C	97.0 (3)
H20A—C20A—H20B	108.8	C19B—C20B—H20C	110.6
C20A—C21A—C17A	103.98 (12)	C21B—C20B—H20C	110.2
C20A—C21A—H21A	111.0	C19C—C20B—H20C	137.9
C17A—C21A—H21A	111.0	C19B—C20B—H20D	110.3
C20A—C21A—H21B	111.0	C21B—C20B—H20D	110.4
C17A—C21A—H21B	111.0	C19C—C20B—H20D	90.0
H21A—C21A—H21B	109.0	H20C—C20B—H20D	108.6
O5A—C22A—N3A	123.32 (14)	C20B—C21B—C17B	105.29 (13)
O5A—C22A—C17A	119.38 (13)	C20B—C21B—H21C	110.7
N3A—C22A—C17A	117.21 (13)	C17B—C21B—H21C	110.7
N3A—C23A—C28A	111.96 (13)	C20B—C21B—H21D	110.7
N3A—C23A—C24A	110.06 (13)	C17B—C21B—H21D	110.7
C28A—C23A—C24A	110.42 (14)	H21C—C21B—H21D	108.8
N3A—C23A—H23A	108.1	O5B—C22B—N3B	122.59 (14)
C28A—C23A—H23A	108.1	O5B—C22B—C17B	119.66 (14)
C24A—C23A—H23A	108.1	N3B—C22B—C17B	117.71 (13)
C25A—C24A—C23A	110.56 (14)	N3B—C23B—C24B	110.78 (13)
C25A—C24A—H24B	109.5	N3B—C23B—C28B	111.91 (13)
C23A—C24A—H24B	109.5	C24B—C23B—C28B	110.06 (13)
C25A—C24A—H24C	109.5	N3B—C23B—H23B	108.0
C23A—C24A—H24C	109.5	C24B—C23B—H23B	108.0
H24B—C24A—H24C	108.1	C28B—C23B—H23B	108.0
C26A—C25A—C24A	110.86 (15)	C23B—C24B—C25B	109.54 (13)
C26A—C25A—H25A	109.5	C23B—C24B—H24A	109.8
C24A—C25A—H25A	109.5	C25B—C24B—H24A	109.8
C26A—C25A—H25B	109.5	C23B—C24B—H24D	109.8
C24A—C25A—H25B	109.5	C25B—C24B—H24D	109.8
H25A—C25A—H25B	108.1	H24A—C24B—H24D	108.2
C27A—C26A—C25A	111.34 (15)	C26B—C25B—C24B	110.44 (13)
C27A—C26A—H26A	109.4	C26B—C25B—H25C	109.6
C25A—C26A—H26A	109.4	C24B—C25B—H25C	109.6
C27A—C26A—H26B	109.4	C26B—C25B—H25D	109.6
C25A—C26A—H26B	109.4	C24B—C25B—H25D	109.6
H26A—C26A—H26B	108.0	H25C—C25B—H25D	108.1
C26A—C27A—C28A	111.77 (15)	C25B—C26B—C27B	111.46 (14)
C26A—C27A—H27A	109.3	C25B—C26B—H26C	109.3
C28A—C27A—H27A	109.3	C27B—C26B—H26C	109.3
C26A—C27A—H27B	109.3	C25B—C26B—H26D	109.3
C28A—C27A—H27B	109.3	C27B—C26B—H26D	109.3
H27A—C27A—H27B	107.9	H26C—C26B—H26D	108.0
C23A—C28A—C27A	110.50 (15)	C26B—C27B—C28B	112.36 (15)
C23A—C28A—H28A	109.5	C26B—C27B—H27C	109.1
C27A—C28A—H28A	109.5	C28B—C27B—H27C	109.1
C23A—C28A—H28B	109.5	C26B—C27B—H27D	109.1
C27A—C28A—H28B	109.5	C28B—C27B—H27D	109.1
H28A—C28A—H28B	108.1	H27C—C27B—H27D	107.9
C2B—O1B—N1B	108.16 (11)	C23B—C28B—C27B	110.37 (14)
C3B—N1B—O1B	111.39 (12)	C23B—C28B—H28C	109.6
C3B—N1B—C4B	135.91 (13)	C27B—C28B—H28C	109.6
O1B—N1B—C4B	111.52 (11)	C23B—C28B—H28D	109.6
C10B—N2B—C11B	121.39 (13)	C27B—C28B—H28D	109.6
C10B—N2B—H2BA	119.3	H28C—C28B—H28D	108.1
C11B—N2B—H2BA	119.3	H1WA—O1W—H1WB	106 (2)
C22B—N3B—C23B	122.75 (13)		
			
C2A—O1A—N1A—C3A	4.14 (16)	C17B—C1B—C2B—O2B	47.6 (2)
C2A—O1A—N1A—C4A	176.37 (12)	C3B—C1B—C2B—O1B	−10.92 (16)
N1A—O1A—C2A—O2A	−179.72 (13)	C17B—C1B—C2B—O1B	−136.68 (13)
N1A—O1A—C2A—C1A	3.76 (15)	O1B—N1B—C3B—O3B	171.91 (14)
C3A—C1A—C2A—O2A	174.92 (16)	C4B—N1B—C3B—O3B	5.9 (3)
C17A—C1A—C2A—O2A	48.5 (2)	O1B—N1B—C3B—C1B	−10.74 (16)
C3A—C1A—C2A—O1A	−9.18 (15)	C4B—N1B—C3B—C1B	−176.79 (15)
C17A—C1A—C2A—O1A	−135.65 (13)	C2B—C1B—C3B—O3B	−169.86 (15)
O1A—N1A—C3A—O3A	173.50 (14)	C17B—C1B—C3B—O3B	−43.6 (2)
C4A—N1A—C3A—O3A	3.7 (3)	C2B—C1B—C3B—N1B	12.87 (15)
O1A—N1A—C3A—C1A	−10.09 (16)	C17B—C1B—C3B—N1B	139.12 (13)
C4A—N1A—C3A—C1A	−179.88 (15)	C3B—N1B—C4B—C5B	−143.84 (17)
C2A—C1A—C3A—O3A	−172.24 (15)	O1B—N1B—C4B—C5B	50.13 (16)
C17A—C1A—C3A—O3A	−45.4 (2)	C3B—N1B—C4B—C10B	93.8 (2)
C2A—C1A—C3A—N1A	11.48 (15)	O1B—N1B—C4B—C10B	−72.23 (15)
C17A—C1A—C3A—N1A	138.28 (13)	C3B—N1B—C4B—C9B	−24.3 (2)
C3A—N1A—C4A—C5A	−144.62 (17)	O1B—N1B—C4B—C9B	169.71 (11)
O1A—N1A—C4A—C5A	45.64 (16)	N1B—C4B—C5B—C6B	66.97 (17)
C3A—N1A—C4A—C10A	93.99 (19)	C10B—C4B—C5B—C6B	−169.16 (14)
O1A—N1A—C4A—C10A	−75.75 (15)	C9B—C4B—C5B—C6B	−52.95 (18)
C3A—N1A—C4A—C9A	−25.8 (2)	C4B—C5B—C6B—C7B	56.25 (19)
O1A—N1A—C4A—C9A	164.48 (11)	C5B—C6B—C7B—C8B	−56.50 (19)
N1A—C4A—C5A—C6A	63.36 (16)	C6B—C7B—C8B—C9B	55.40 (19)
C10A—C4A—C5A—C6A	−173.85 (13)	C7B—C8B—C9B—C4B	−54.89 (19)
C9A—C4A—C5A—C6A	−56.01 (17)	N1B—C4B—C9B—C8B	−66.83 (17)
C4A—C5A—C6A—C7A	56.96 (17)	C5B—C4B—C9B—C8B	52.77 (18)
C5A—C6A—C7A—C8A	−54.57 (18)	C10B—C4B—C9B—C8B	171.30 (13)
C6A—C7A—C8A—C9A	53.78 (18)	C11B—N2B—C10B—O4B	3.4 (2)
C7A—C8A—C9A—C4A	−55.29 (17)	C11B—N2B—C10B—C4B	−172.06 (13)
N1A—C4A—C9A—C8A	−63.82 (16)	N1B—C4B—C10B—O4B	161.28 (14)
C5A—C4A—C9A—C8A	55.27 (16)	C5B—C4B—C10B—O4B	39.4 (2)
C10A—C4A—C9A—C8A	174.07 (12)	C9B—C4B—C10B—O4B	−78.65 (18)
C11A—N2A—C10A—O4A	0.1 (2)	N1B—C4B—C10B—N2B	−23.0 (2)
C11A—N2A—C10A—C4A	−178.09 (13)	C5B—C4B—C10B—N2B	−144.92 (15)
N1A—C4A—C10A—O4A	167.89 (13)	C9B—C4B—C10B—N2B	97.08 (16)
C5A—C4A—C10A—O4A	46.77 (19)	C10B—N2B—C11B—C12B	−160.56 (14)
C9A—C4A—C10A—O4A	−71.47 (18)	C10B—N2B—C11B—C16B	76.50 (19)
N1A—C4A—C10A—N2A	−13.82 (19)	N2B—C11B—C12B—C13B	179.36 (13)
C5A—C4A—C10A—N2A	−134.94 (15)	C16B—C11B—C12B—C13B	−57.53 (18)
C9A—C4A—C10A—N2A	106.82 (15)	C11B—C12B—C13B—C14B	58.15 (19)
C10A—N2A—C11A—C12A	−158.56 (14)	C12B—C13B—C14B—C15B	−57.6 (2)
C10A—N2A—C11A—C16A	78.39 (18)	C13B—C14B—C15B—C16B	56.3 (2)
N2A—C11A—C12A—C13A	179.65 (13)	C14B—C15B—C16B—C11B	−55.4 (2)
C16A—C11A—C12A—C13A	−56.80 (18)	N2B—C11B—C16B—C15B	178.77 (14)
C11A—C12A—C13A—C14A	56.2 (2)	C12B—C11B—C16B—C15B	56.24 (19)
C12A—C13A—C14A—C15A	−55.4 (2)	C2B—C1B—C17B—C22B	59.88 (17)
C13A—C14A—C15A—C16A	55.9 (2)	C3B—C1B—C17B—C22B	−59.04 (17)
N2A—C11A—C16A—C15A	179.15 (14)	C2B—C1B—C17B—C18B	−174.71 (13)
C12A—C11A—C16A—C15A	56.66 (19)	C3B—C1B—C17B—C18B	66.37 (17)
C14A—C15A—C16A—C11A	−56.4 (2)	C2B—C1B—C17B—C21B	−62.39 (17)
C2A—C1A—C17A—C22A	61.15 (16)	C3B—C1B—C17B—C21B	178.69 (13)
C3A—C1A—C17A—C22A	−58.46 (16)	C22B—C17B—C18B—C19C	−127.4 (4)
C2A—C1A—C17A—C18A	−172.21 (12)	C1B—C17B—C18B—C19C	112.1 (4)
C3A—C1A—C17A—C18A	68.18 (16)	C21B—C17B—C18B—C19C	−6.0 (4)
C2A—C1A—C17A—C21A	−61.23 (17)	C22B—C17B—C18B—C19B	−158.39 (17)
C3A—C1A—C17A—C21A	179.15 (12)	C1B—C17B—C18B—C19B	81.06 (19)
C22A—C17A—C18A—C19A	−158.17 (14)	C21B—C17B—C18B—C19B	−37.00 (19)
C1A—C17A—C18A—C19A	80.39 (16)	C19C—C18B—C19B—C20B	−79.9 (7)
C21A—C17A—C18A—C19A	−36.66 (16)	C17B—C18B—C19B—C20B	24.8 (2)
C17A—C18A—C19A—C20A	17.36 (19)	C19B—C18B—C19C—C20B	61.7 (7)
C18A—C19A—C20A—C21A	9.37 (19)	C17B—C18B—C19C—C20B	−23.0 (5)
C19A—C20A—C21A—C17A	−32.55 (17)	C18B—C19B—C20B—C21B	−2.1 (3)
C22A—C17A—C21A—C20A	166.66 (13)	C18B—C19B—C20B—C19C	71.2 (6)
C1A—C17A—C21A—C20A	−74.24 (15)	C18B—C19C—C20B—C19B	−69.4 (6)
C18A—C17A—C21A—C20A	42.73 (15)	C18B—C19C—C20B—C21B	43.0 (4)
C23A—N3A—C22A—O5A	−5.2 (2)	C19B—C20B—C21B—C17B	−21.4 (2)
C23A—N3A—C22A—C17A	171.36 (13)	C19C—C20B—C21B—C17B	−48.7 (3)
C1A—C17A—C22A—O5A	−5.97 (18)	C22B—C17B—C21B—C20B	159.15 (14)
C18A—C17A—C22A—O5A	−129.38 (15)	C1B—C17B—C21B—C20B	−82.06 (16)
C21A—C17A—C22A—O5A	115.35 (15)	C18B—C17B—C21B—C20B	36.00 (16)
C1A—C17A—C22A—N3A	177.34 (13)	C23B—N3B—C22B—O5B	−1.9 (2)
C18A—C17A—C22A—N3A	53.93 (18)	C23B—N3B—C22B—C17B	175.88 (14)
C21A—C17A—C22A—N3A	−61.34 (18)	C1B—C17B—C22B—O5B	−5.3 (2)
C22A—N3A—C23A—C28A	100.86 (18)	C18B—C17B—C22B—O5B	−128.31 (16)
C22A—N3A—C23A—C24A	−135.92 (15)	C21B—C17B—C22B—O5B	116.19 (16)
N3A—C23A—C24A—C25A	177.65 (14)	C1B—C17B—C22B—N3B	176.90 (14)
C28A—C23A—C24A—C25A	−58.23 (18)	C18B—C17B—C22B—N3B	53.86 (19)
C23A—C24A—C25A—C26A	57.0 (2)	C21B—C17B—C22B—N3B	−61.63 (19)
C24A—C25A—C26A—C27A	−55.1 (2)	C22B—N3B—C23B—C24B	−135.46 (15)
C25A—C26A—C27A—C28A	54.6 (2)	C22B—N3B—C23B—C28B	101.30 (17)
N3A—C23A—C28A—C27A	−179.83 (14)	N3B—C23B—C24B—C25B	174.80 (13)
C24A—C23A—C28A—C27A	57.15 (19)	C28B—C23B—C24B—C25B	−60.90 (17)
C26A—C27A—C28A—C23A	−55.5 (2)	C23B—C24B—C25B—C26B	59.43 (17)
C2B—O1B—N1B—C3B	3.71 (16)	C24B—C25B—C26B—C27B	−55.29 (19)
C2B—O1B—N1B—C4B	173.33 (12)	C25B—C26B—C27B—C28B	52.8 (2)
N1B—O1B—C2B—O2B	−178.50 (14)	N3B—C23B—C28B—C27B	−178.54 (13)
N1B—O1B—C2B—C1B	5.15 (16)	C24B—C23B—C28B—C27B	57.82 (18)
C3B—C1B—C2B—O2B	173.34 (17)	C26B—C27B—C28B—C23B	−53.88 (19)

### Hydrogen-bond geometry (Å, °)

**Table:**

*D*—H···*A*	*D*—H	H···*A*	*D*···*A*	*D*—H···*A*
N2A—H2AA···O5A	0.88	2.09	2.9371 (18)	161
N3A—H3AA···O1W	0.88	2.00	2.8662 (19)	170
N2B—H2BA···O5B	0.88	2.10	2.9330 (18)	157
N3B—H3BA···O3A	0.88	2.07	2.9238 (17)	163
O1W—H1WA···O4B^i^	0.85 (3)	1.90 (3)	2.7433 (18)	169 (2)
O1W—H1WB···O4A^ii^	0.89 (2)	1.88 (2)	2.7569 (18)	170 (2)

Symmetry codes: (i) −*x*+1, −*y*+2, −*z*+1; (ii) *x*+1, *y*, *z*.
